# Basic safety first: trauma-informed care in a hostile environment

**DOI:** 10.1192/bjb.2019.91

**Published:** 2020-04

**Authors:** Cate Bailey

**Affiliations:** 1 East London NHS Foundation Trust, UK; 2 Barts and the London School of Medicine, UK

**Keywords:** Trauma, post-traumatic stress disorder, asylum seeker, refugees

## Abstract

This editorial introduces and reflects on a Praxis article in the trainees' section of this issue. The article, 'Assessing asylum seekers, refugees and undocumented migrants' by Waterman *et al*, begins with a clinical scenario describing an emergency presentation at a ‘place of safety’. The authors are to be congratulated for navigating a compassionate path through the complexities of law, health and new diagnostic categories. The resources found in the article, drawing on the principles of trauma-informed care and the work of Judith Herman, can help trainees to be more confident in promoting the basic rights of survivors of trauma, which might form a first step in the re-establishment of trust and empowerment.

There has never been a more timely moment to highlight the needs and rights of asylum seekers and refugees, as so comprehensively described in this month's Praxis article ‘Assessing asylum seekers, refugees and undocumented migrants’, by Waterman *et al*.^1^ This population and those who care for them face many challenges. Not least of these is the ongoing hostile environment. In the UK, immigration checks are embedded in everyday interactions and accessing healthcare may result in sharing of personal data with the Home Office, leading to deportation or detention.^2^ Frequently changing funding arrangements related to Brexit threaten numerous charities that support migrants.^3^ However, Waterman *et al*'s article can empower us to be hopeful and compassionate and, importantly, well-versed in relevant law. In so doing it embraces some of the core values of a psychiatrist as outlined by the Royal College of Psychiatrists, including advocacy, empowerment and a person-centred biopsychosocial approach.^4^

The article introduces a fictitious but sadly all too familiar case, which begins with a presentation in crisis at an inner-city ‘place of safety’. What I have learned in reviewing this article has already helped me to advocate for two patients under my care to receive National Health Service (NHS) treatment and eligibility for housing that was called into question owing to legal uncertainties. Both had been the victims of torture and were at varying stages in asylum claims. The securing of such basic rights is the first step for survivors of trauma beginning to find safety and regain control over their lives, what Judith Herman describes as a primary stage in her book *Trauma and Recovery* (p. 326).^5^ However, it would seem that I was not alone in my ignorance of the complexities of law. A recent survey of a 514 health professionals found that only 26% were aware that all migrants were entitled to free general practitioner (GP) services.^6^ Just 39% were able to identify which groups of migrants were entitled to non-emergency NHS care.^6^

## Individual and societal responses to trauma

In words that still hold true today, Herman wrote in the introduction to her powerful work: ‘The ordinary response to atrocities is to banish them from consciousness’.^5^ Psychiatry and trauma have a long and complicated relationship. Trauma has had an often ‘underground’ history, resurfacing and then disappearing at various points depending on the sociopolitical climate.^7^ As far back as 1919 Janet recognised that the survivors of trauma ‘are unable to make the recital which we call narrative memory, and yet they remain confronted by [the] difficult situation’ (Janet 1919: see van der Kolk *et al*^8^). Years later Kardiner built on Janet's studies of ‘hysteria’ in women to formulate the outlines of traumatic syndromes in male combat veterans (Kardiner 1941: see van der Kolk *et al*^8^). Contemporary psychiatrists such as Herman and van der Kolk have formulated and communicated clearly and conclusively how traumatic life experiences have far-reaching effects on the mind and body. This has paved the way for an understanding of the neurobiological effects of trauma on the brain, endocrine and immune systems, including dysregulation of the hypothalamic–pituitary–adrenal axis and sympathetic nervous hyperarousal.^9^ Thanks to Felitti and colleagues’ large and important study on adverse childhood experiences (ACEs), there are now robust and undeniable links between exposure to trauma and cardiovascular and respiratory disease, cancer, obesity, chronic pain and gastrointestinal illness.^9,10^ The intergenerational effects of trauma are also being increasingly understood. It has recently been found that the children of mothers who were Holocaust survivors and suffered post-traumatic stress disorder (PTSD) have increased glucocorticoid sensitivity.^11^

Herman, more than 20 years ago, identified the syndrome of complex post-traumatic stress disorder (CPTSD).^12^ Yet it has taken tireless work and campaigning from clinicians, researchers and people with lived experience for it to now be included in ICD-11.^13^ The operationalisation and recognition of the diagnosis is a significant step towards research and treatments which acknowledge that prolonged and repeated trauma profoundly affects the sense of self, affective stability and relationships. Indeed, some studies have shown that CPTSD may be even more common that PTSD,^13^ and deserves specialist attention, both within services and also in the educational curricula for future psychiatrists.

## Facing a culture of disbelief

The article by Waterman and colleagues avoids the scapegoating of refugees^14^ and the ‘denial, repression and dissociation’ of trauma that Herman identified operating on both societal and individual levels.^5^ Refugees and asylum seekers who are survivors of trauma encounter multiple challenges, which are described in the article. First, there is the trauma itself, which is known to cause ‘disintegration of experience’ and loss of narrative.^8^ Echoing Janet's work, Herman describes how ‘people who have survived atrocities often tell their stories in a highly emotional, contradictory and fragmented manner’.^5^ Yet the processes of accessing healthcare or asylum demand that survivors tell their story repeatedly, and they are received by a ‘culture of disbelief’ and face both testimonial and hermeneutical injustice.^2,15^ Individuals who are already hypervigilant to threat must navigate health services that may share information with the Home Office, mistakenly charge them for treatment and inadvertently re-victimise them with unjust, uneducated and sometimes perverse decisions.^2^ Psychiatrist Sandra Bloom states: ‘because of complex interactions between traumatized clients, stressed staff, pressured organizations, and a social and economic environment that is frequently hostile to the aims of recovery, our systems frequently recapitulate the very experiences that have proven to be so toxic for the people we are supposed to treat’.^16^

## Trauma-informed and human rights-based care

Trauma-informed care can provide some framework for individuals and health services and is premised on a fundamental shift from thinking ‘What's wrong with you?’ to ‘What happened to you?’.^17^ Such approaches seek to rebuild trust, create safe environments, empower and to avoid re-traumatisation.^17^

After reading this Praxis article I would urge trainees to familiarise themselves with the principles of trauma-informed care and to think how these might be applied in their organisations. Sweeney and colleagues’ article in *BJPsych Advances* is an extremely helpful summary.^17^ We must also remember that trauma does not occur only in wars or in other countries; experiences of torture and childhood adversity are all too common, as highlighted by the original study of ACEs in the USA.^10^ Research in the UK has found a similarly high frequency, with 47% of almost 4000 participants reporting having experienced at least one ACE.^18^ The pivotal works of Herman^5^ and van der Kolk^19^ provide a compelling, compassionate and eloquent narrative on a subject that is so often unspeakable. Practical resources and links to specialist agencies for asylum seekers and refugees can be found as an online supplement to the Praxis article.^1^ Trainees may be interested to explore the various communities of healthcare professionals who advocate for human rights-based approaches to healthcare such as Medact (www.medact.org/project/migration-health), Docs Not Cops (www.docsnotcops.co.uk) and the Twitter hashtag #patientsnotpassports. Poetry and storytelling can also communicate and transform, with the potential to both educate and liberate from shame and secrecy.

British-Somali poet Warsan Shire writes in her poem ‘Home’:

‘no one leaves home unless
home is the mouth of a shark.
You only run for the border
when you see the whole city
running as well.’^20^

I hope that Waterman and colleagues’ article offers trainees a framework for thinking about providing safe and compassionate care to asylum seekers, refugees and undocumented migrants. I thank the authors for their perseverance in bringing together complex legislation and issues of mental and physical healthcare in such a fine example of what we are seeking in Praxis articles. The article and the work of Herman, van der Kolk and the principles of trauma-informed care demand us as clinicians, as fellow humans, to bear witness and to empower survivors. We must strive to avoid re-traumatisation and rejection, despite the current hostile environment in which we live and practice.

## About the author

**Cate Bailey**, MBBS, MRCPsych, MRes, is a Fellow in Medical Education at East London NHS Foundation Trust; an honorary clinical lecturer at Barts and the London School of Medicine; Trainees’ Section Editor of the *BJPsych Bulletin*; and Co-chair of the Older Adults Section of the Association for Psychoanalytic Psychotherapy in the NHS, UK.
